# Transcriptome Profiling of Resistance to *Fusarium oxysporum* f. sp. *conglutinans* in Cabbage (*Brassica oleracea*) Roots

**DOI:** 10.1371/journal.pone.0148048

**Published:** 2016-02-05

**Authors:** Miaomiao Xing, Honghao Lv, Jian Ma, Donghui Xu, Hailong Li, Limei Yang, Jungen Kang, Xiaowu Wang, Zhiyuan Fang

**Affiliations:** 1 Beijing Vegetable Research Center, Beijing Academy of Agriculture and Forestry Sciences, Key Laboratory of Biology and Genetic Improvement of Horticultural Crops (North China), Ministry of Agriculture, 50# Zhanghua Street, Beijing 100097, China; 2 Institute of Vegetables and Flowers, Chinese Academy of Agricultural Sciences, Key Laboratory of Biology and Genetic Improvement of Horticultural Crops, Ministry of Agriculture, 12# Zhongguancun Nandajie Street, Beijing 100081, China; The University of Wisconsin - Madison, UNITED STATES

## Abstract

Fusarium wilt caused by *Fusarium oxysporum* f. sp. *conglutinans* (FOC) is a destructive disease of *Brassica* crops, which results in severe yield losses. There is little information available about the mechanism of disease resistance. To obtain an overview of the transcriptome profiles in roots of R4P1, a *Brassica oleracea* variety that is highly resistant to fusarium wilt, we compared the transcriptomes of samples inoculated with FOC and samples inoculated with distilled water. RNA-seq analysis generated more than 136 million 100-bp clean reads, which were assembled into 62,506 unigenes (mean size = 741 bp). Among them, 49,959 (79.92%) genes were identified based on sequence similarity searches, including SwissProt (29,050, 46.47%), Gene Ontology (GO) (33,767, 54.02%), Clusters of Orthologous Groups (KOG) (14,721, 23.55%) and Kyoto Encyclopedia of Genes and Genomes Pathway database (KEGG) (12,974, 20.76%) searches; digital gene expression analysis revealed 885 differentially expressed genes (DEGs) between infected and control samples at 4, 12, 24 and 48 hours after inoculation. The DEGs were assigned to 31 KEGG pathways. Early defense systems, including the MAPK signaling pathway, calcium signaling and salicylic acid-mediated hypersensitive response (SA-mediated HR) were activated after pathogen infection. SA-dependent systemic acquired resistance (SAR), ethylene (ET)- and jasmonic (JA)-mediated pathways and the lignin biosynthesis pathway play important roles in plant resistance. We also analyzed the expression of defense-related genes, such as genes encoding pathogenesis-related (PR) proteins, UDP-glycosyltransferase (UDPG), pleiotropic drug resistance, ATP-binding cassette transporters (PDR-ABC transporters), myrosinase, transcription factors and kinases, which were differentially expressed. The results of this study may contribute to efforts to identify and clone candidate genes associated with disease resistance and to uncover the molecular mechanism underlying FOC resistance in cabbage.

## Introduction

Fusarium wilt is a destructive disease that causes great losses to cabbage (*Brassica oleracea* L. var. *capitata*) production worldwide. This disease was first identified in the United States by Smith in the 1890s [1], and in the following decades was subsequently found in Japan and several other countries [2]. In recent years, fusarium wilt has been identified in several provinces in China [3–5].

Cabbage fusarium wilt is a soil-borne disease caused by pathogen FOC (*Fusarium oxysporum* f. sp. *Conglutinans*), which can remain in soil for years or even decades [6]. This pathogen infects cabbage roots, colonizes and occludes the xylem vessels, and leads to leaf wilt or sometimes wilting of the entire plant, with stunted growth and eventually death. Traditional methods, such as crop rotation and chemical control, have almost no effect on the disease because this pathogen is ubiquitous in soil and is not eradicated by these methods. Consequently, developing resistant cultivars is considered to be the most effective measure to control fusarium wilt in cabbage [7].

Molecular mapping of genes controlling fusarium wilt resistance has been extensively reported over the past five years. In order to determine the inheritance pattern of resistance to Fusarium in cabbage and to clone FOC resistance genes for marker-assisted selection (MAS) in cabbage resistance breeding, Jiang et al. [8] developed the stable SCAR marker S46M48199, which is linked in repulsion to the dominant allele of the fusarium wilt resistance gene *FOC-1* at a distance of 2.78 cM in cabbage. Pu et al. [7] mapped fusarium wilt resistance gene *Foc-Bo1* to linkage group seven (O7) using both segregation test and quantitative trait locus (QTL) analysis in cabbage, and they eventually cloned *FocBo1*, which encodes a TIR-NBS-LRR type R gene [9]. Lv et al. [10] developed two InDel markers, M10 and A1, flanking the FOC resistance gene at 1.2 and 0.6 cM, respectively, based on a DH population, ultimately mapping the candidate resistance gene to Bol037156 on chromosome C06; this gene encodes a putative TIR-NBS-LRR type R protein [11]. However, these reports do not provide a comprehensive view of the defense mechanism to FOC in cabbage.

High-throughput RNA sequencing (RNA-seq) technology represents a powerful and efficient method for transcriptome analysis and has led to the discovery of many interesting genes. It has made it possible to monitor disease resistance-related gene expression profiles and to reveal the signal transduction pathways involved in the defense network. Applying genome wide transcriptomics to study host-pathogen interactions has provided insights into the mechanisms underlying disease development, basal defense and gene-for-gene resistance. Several transcriptome profiling studies of plants following inoculation with Fusarium fungus have been reported, including studies in watermelon [12], banana [13], *Arabidopsis thaliana* [14, 15] and wheat [16]. In these studies, the following have been investigated in plant defense responses: defense-related genes, including pathogenesis-related (PR) genes, phytoalexin, transcription factors, protein kinase and ROS-related genes, resistance (*R*) genes; and the roles of salicylic acid (SA), jasmonic acid (JA) and ethylene (ET) signaling pathways. Signaling pathways activating disease resistance are commonly classified as gene-for-gene resistance responses, SA-dependent responses, and JA- and ET-dependent responses. All of these studies suggest that JA signaling plays an important role in resistance to Fusarium. Some studies have shown that SA signaling is not involved in resistance to necrotrophic pathogens or necrotrophic phases [13, 15, 16]. However, a study examining the response to *F*. *oxysporum*-infection in *Arabidopsis* [10] showed that genes associated with SA-dependent SAR were induced at 6 DPI (days post infection), when *F*. *oxysporum* switches from a biotrophic to a necrotrophic lifestyle [16] and when the SA pathway is not activated. Although study showed that effective defense responses against biotrophic pathogens are associated with the activation of the SA-dependent defense pathway, and JA and ET signaling are activated during defense responses to necrotrophic pathogens [17], there are exceptions to this rule, and the situation is complex. The details of the resistance response to FOC in cabbage remain to be elucidated.

In this study, we performed the first global analysis of transcriptome dynamics during the FOC defense response in cabbage using RNA-seq. Specifically, we analyzed the differential gene expression patterns between inoculated and control roots at various time points. The results of this study will help reveal genes and pathways associated with resistance to FOC in cabbage, which contributes to our understanding of the FOC resistance mechanism.

## Materials and Methods

### Preparation of material

The fungal strain GLHW1, isolated from diseased cabbage in Shouyang, Shanxi province, China, which belongs to Race 1 [4, 5] was incubated on potato-dextrose agar plates at 26–28°C for 5–7 days, following by growth in potato-lactose broth on a shaker at 125 rpm at 26–28°C for 5–7 days. The suspension concentration was adjusted to 1×10^6^ spores ml^-1^ with sterile distilled water prior to inoculation.

R4P1 (resistant) and R2P2 (susceptible) seeds were grown in sterilized soil (peat: vermiculite = 1:1) in an artificial climate box at 25°C/18°C day/night temperatures with a 16-h light/8-h dark photoperiod. Seedlings at the three-leaf stage were infected with FOC by root dip inoculation into a suspension of fungal spores for 15 min and then were returned to their original pots, where they were grown at 28°C under the same photoperiod [18]. Control plants were treated in a similar manner but were mock-inoculated with distilled water. Both inoculated and mock-inoculated plant roots were sampled at 4, 12, 24 and 48 hai (hours after inoculation). Five individual seedlings were used per replicate, with a total of three replicates collected per treatment at each sampling time point.

### RNA isolation and cDNA library construction

Total RNAs were isolated using the RNAprep Pure Plant Kit (TIANGEN). Twelve RNA samples isolated from R4P1 roots at 4, 12, 24 and 48 hai, including eight inoculated samples (FW4_1, FW4_2, FW12_1, FW12_2, FW24_1, FW24_2, FW48_1 and FW48_2) and four mock-inoculated samples (DW4, DW12, DW24 and DW48), were sent to Novogene Bioinformatics Technology Co. Ltd. (Beijing) for library construction and sequencing.

RNA integrity was confirmed using the RNA Nano 6000 Assay Kit of the Bioanalyzer 2100 system (Agilent Technologies, CA, USA) with a minimum RNA integrated number of 8. Sequencing libraries were generated using an Illumina TruSeq^™^ RNA Sample Preparation Kit (Illumina, San Diego, CA, USA). Briefly, mRNA was purified from 3 μg total RNA using poly-T oligo-attached magnetic beads and fragmented using Illumina proprietary fragmentation buffer at 94°C for 15 min. First-strand cDNA was synthesized using a 6 bp-random primer and SuperScript II. Second-strand cDNA was then synthesized. These cDNA fragments were then subjected to an end repair process, and to adenylation and ligation of adapters P5 and P7. The insert size of the library was restricted to approximately 300 bp using PCR products purified with the AMPure XP system (Beckman Coulter, Beverly, MA, USA).

### Illumina sequencing and assembly for transcriptome analysis

Transcriptome sequencing generated 100 bp paired-end (PE) raw reads using the Illumina HiSeq 2000. The raw reads were then filtered by discarding adaptor reads, reads with the ratio of ‘N’ >10% and low quality reads with more than 50% bases of quality value ≤5. The remaining clean reads were then assembled using Trinity software for transcriptome assembly without a reference genome, with min_kmer_cov set to 2 by default and all other parameters set to default values [19].

Homology annotations were performed using the following public databases: NCBI Nr (non-redundant protein), NCBI Nt (nucleotide sequences), Swiss-Prot, GO (Gene Ontology), KOG (euKaryotic Ortholog Groups) and KEGG (Kyoto Encyclopedia of Genes and Genomes). If the results from different database searches conflicted, they were prioritized in the following order: Nr, Nt, KEGG, Swiss-Prot, GO and KOG. The threshold values for Nr, Nt and Swiss-Prot were 1e-5; that for KOG was 1e^-3^. And the genome data of *Arabidopsis thaliana* [20, 21], *Brassica rapa* [22] and *Brassica oleracea* [23] were also used for the analysis of unigenes.

### Illumina sequencing and mapping for differential gene expression analysis

The SE100 (single-end) sequencing strategy was used for digital gene expression profiling. All clean reads were mapped to the assembled reference sequences with a restriction of no more than 1 bp mismatch. Mapping was conducted using RSEM software [24]. The number of mapped clean reads was calculated and normalized to RPKM values [25]. Read count values are input data used to analyze differential gene expression. For samples with biological replicates, differential expression analysis of two samples was performed using the DESeq R package [26]; genes with an adjusted p-value < 0.05 by DESeq were determined to be differentially expressed. For samples without biological replicates, the DEGseq R package [27] was used; q-value < 0.005 and |log2 (fold change) |>1 were set as the thresholds for differential gene expression. RPKM was used for subsequent analysis, such as correlation analysis of gene expression and DEG cluster analysis.

GO enrichment analysis of the differentially expressed genes (DEGs) was implemented by the GOseq R packages based on Wallenius’ noncentral hypergeometric distribution [28], which can adjust for gene length bias in DEGs. KOBAS [29] software was used to test the statistical enrichment of DEGs in KEGG pathways.

### Validation of DEGs by quantitative RT-PCR

DEGs selected were validated using qPCR. The cDNA was synthesized from the same samples used for Illumina sequencing. *GAPDH* was used as an internal reference. RT-PCR was performed using SYBR Green 1 (TIANGEN) on a LightCycler 480 II Real-Time PCR Detection System (Bio-Rad, USA). The reaction was carried out in a total volume of 20 μL containing 10 μL of 2× SuperReal PreMix Plus, 2 μL of cDNA mix, 0.6 μL of each primer and 6.8 μL of RNase-free ddH_2_O. The thermal profile for RT-PCR was 95°C for 15 min, followed by 40 cycles of 95°C for 10 s, 60°C for 20 s and 72°C for 20 s. Melting curve analysis was performed at the end of each PCR reaction at 95°C for 5 s, 65°C for 60 s, 97°C (continuous), 40°C for 30 s. Relative expression was calculated using the comparative CT method (2^-ΔΔCt^ method). Genes and primer sequences can be found in S1 Table.

## Results

### Transcriptome characterization of R4P1 roots after infection with FOC by high-throughput RNA sequencing

All inoculated and non-inoculated samples were equally mixed (designated Trans_BO) and sequenced for transcriptome analysis. After raw read filtering and quality checks, more than 136 million 100-bp clean reads were obtained. Transcriptome assembly was carried out with Trinity software, resulting in the identification of 62,506 unigenes with an average length of 741 bp for annotation (Table 1), total of 49,959 unigenes (79.92% of all unigenes) were identified in at least one database.

**Table 1:** Statistical results of unigene annotations.

Database type	Number of unigenes	Percentage (%)
NT	34036	54.45
NR	43501	69.59
SwissProt	29050	46.47
PFAM	25716	41.14
KO	12974	20.75
GO	33767	54.02
KOG	14721	23.55
Annotated in at least one Database	49959	79.92
Total Unigenes	62506	100

**Fig 1:**
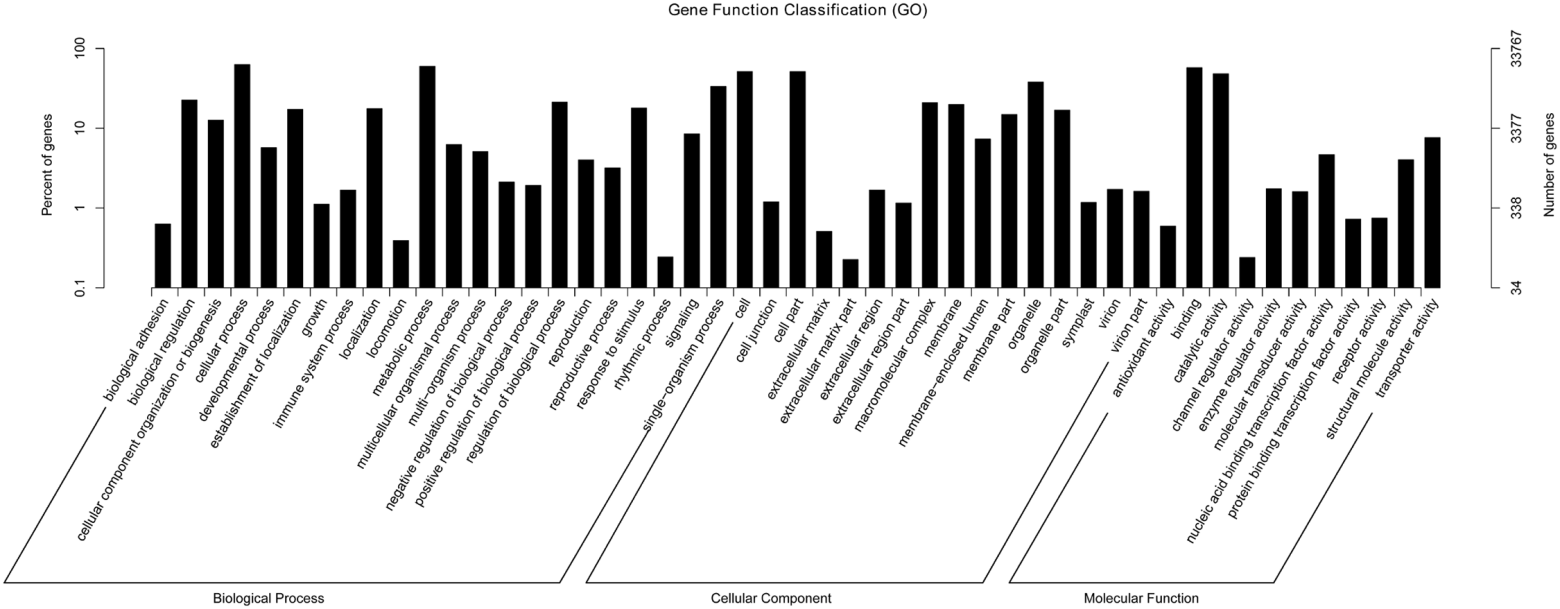
Gene ontology classification of unigenes.

GO enrichment analysis was performed to classify gene functions. A total of 33,767 unigenes were assigned to 49 GO terms in three categories: BP (Biological process), CC (Cellular component) and MF (Molecular Function). A high proportion of DEGs were assigned to cellular process and metabolic process in the BP category, to cell, cell part and organelle in CC and to binding and catalytic activity in MF (Fig 1).

**Fig 2:**
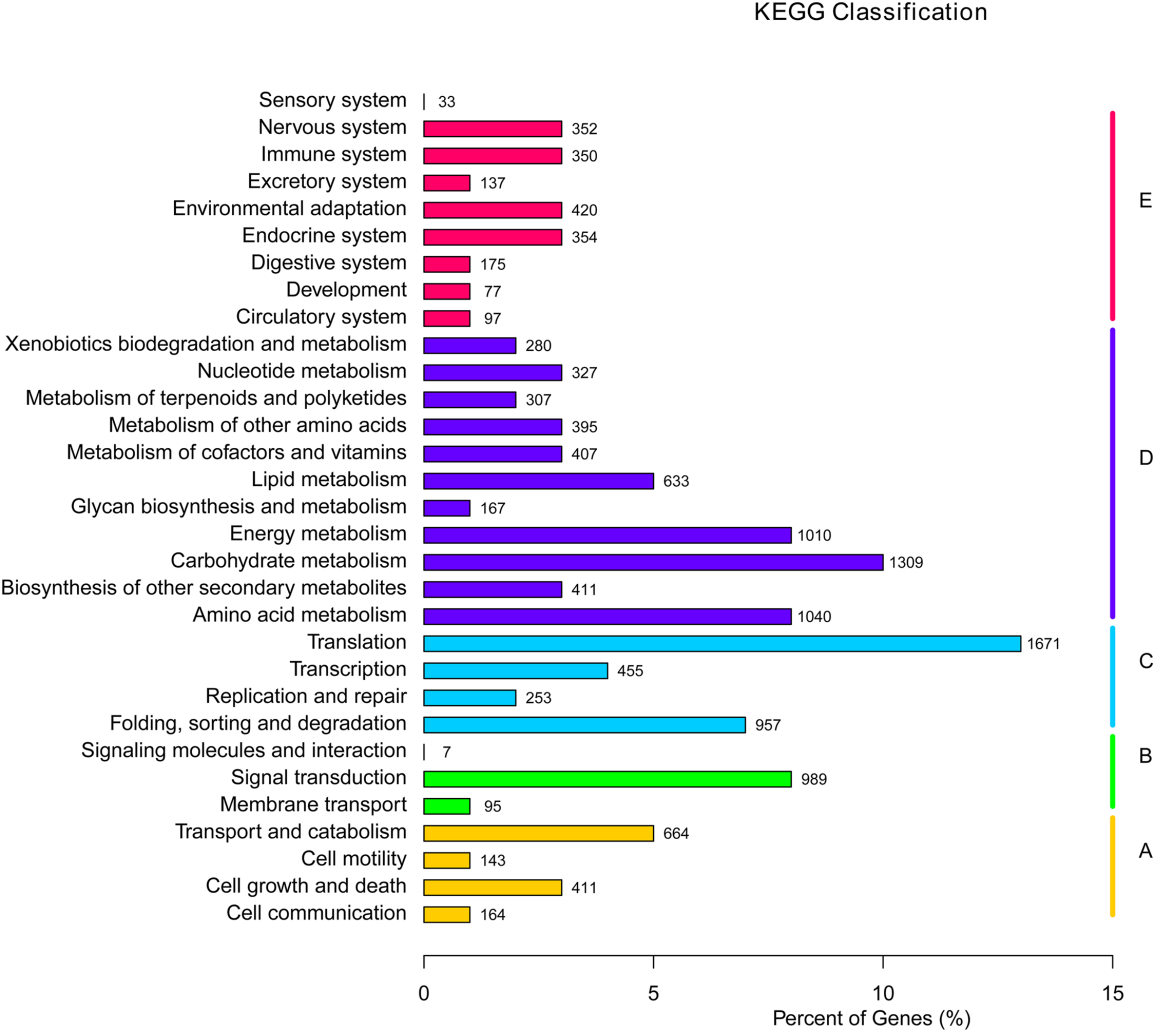
KEGG classification of unigenes. (A) Cellular Processes. (B) Environmental Information Processing. (C) Genetic Information Processing. (D) Metabolism. (E) Organismal Systems.

A total of 12,974 unigenes were annotated in GO and were then classified in the KEGG database to analyze the metabolic pathways in which they participate, which were divided into five branches: A (Cellular Processes), B (Environmental Information Processing), C (Genetic Information Processing), D (Metabolism) and E (Organismal Systems). In each of the five branches, the most highly represented pathways were environmental adaption (420, 0.67%), carbohydrate metabolism (1,309, 2.09%), translation (1,671, 2.67%), signal transduction (989, 1.58%) and transport and catabolism (664, 1.06%). Moreover, 350 unigenes (0.56% of all unigenes) participate in the immune system (Fig 2).

**Fig 3:**
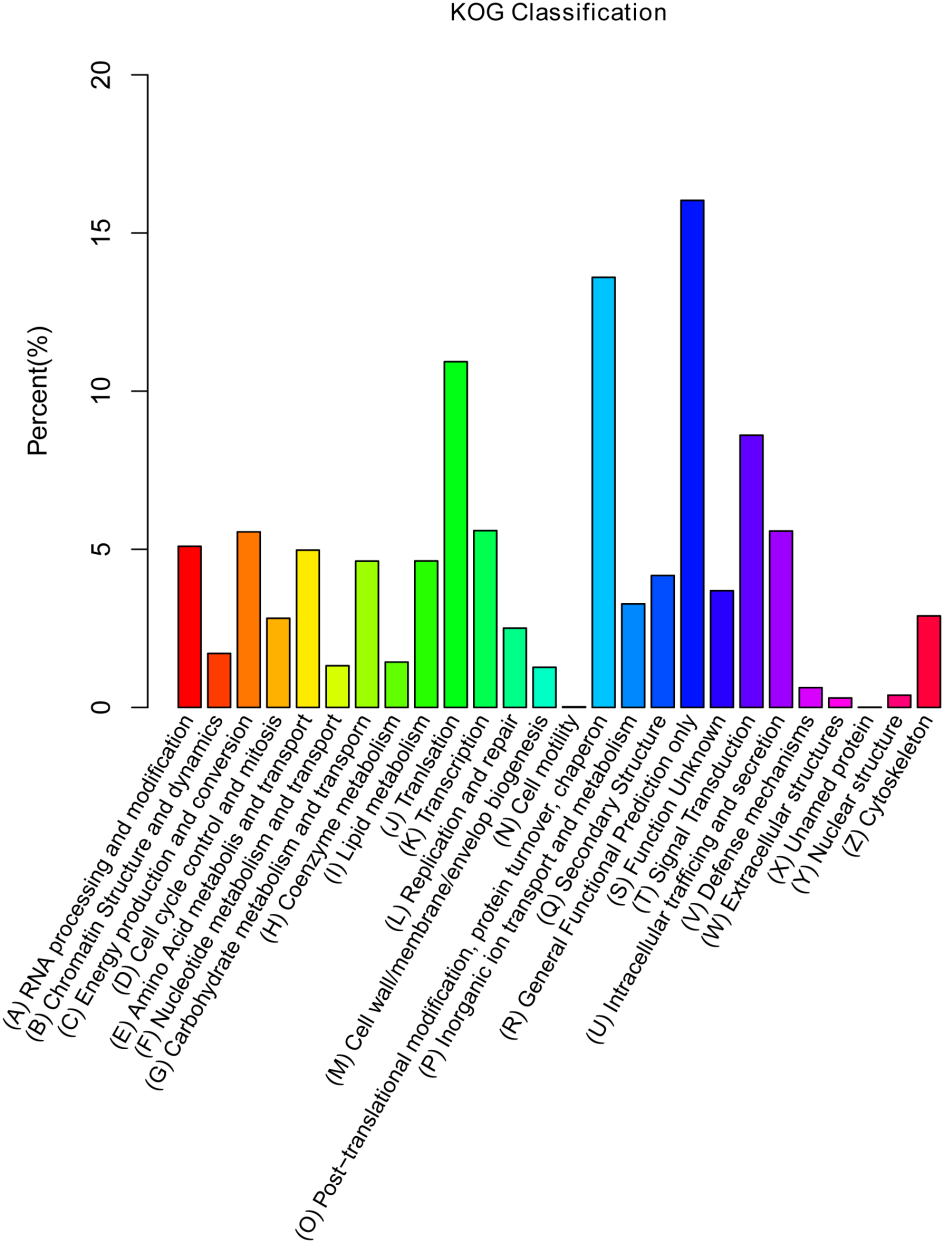
KOG classification.

In addition, 14,721 unigenes (23.55%) were assigned to 26 KOG functional categories (Fig 3). Among these genes, ‘general function prediction only’ represents the largest group (2,360, 3.77%), followed by ‘post-translational modification, protein turnover, and chaperones’ (2,002, 3.20%), ‘translation’ (1,609, 2.57%) and ‘signal transduction’ (1,267, 2.03%).

### Digital gene expression library sequencing and annotation

Based on the transcriptome data, 12 libraries were constructed to identify the gene expression profiles of R4P1 roots during FOC infection, including DW4, FW4_1, FW4_2, DW12, FW12_1, FW12_2, DW24, FW24_1, FW24_2, DW48, FW48_1 and FW48_2 (table 2). Each library generated 10.85 to 15.74 million raw reads. After performing a quality check, the number of clean reads ranged from 10.72 to 15.52 million. The minimum and maximum GC percent values of the 12 libraries were 45.72% and 46.70%, respectively. The Q20 percentages were ≥96.50% and the number of reads mapped to 62,506 unigenes using RSEM ranged from 13,832,709 (89.08%) to 13,469,925 (91.13%) [24], confirming that the sequencing data were appropriate for subsequent analysis.

**Table 2:** Data quality evaluation of sample.

Sample name	Raw reads	Clean reads	GC (%)	Error (%)	Q20 (%)	Total mapped
DW4	14828646	14638472	46.28	0.04	96.54	13339188(91.12%)
FW4_1	11124834	10985291	46.31	0.04	96.64	9970094 (90.76%)
FW4_2	13629475	13435056	46.55	0.04	96.55	12162626(90.53%)
DW12	14950551	14780536	45.72	0.04	96.87	13469925(91.13%)
FW12_1	14905611	14714091	46.11	0.04	96.84	13383604(90.96%)
FW12_2	10853023	10724071	46.13	0.04	96.58	9603058 (89.55%)
DW24	15461231	15289907	46.51	0.04	96.68	13912721(90.99%)
FW24_1	15748199	15529280	46.45	0.04	96.72	13832709(89.08%)
FW24_2	12921595	12747599	46.01	0.04	96.60	11423440(89.61%)
DW48	11949074	11785699	46.70	0.04	96.54	10655203(90.41%)
FW48_1	11187930	11038671	46.63	0.04	96.58	10023240(90.80%)
FW48_2	15715268	15512388	46.16	0.04	96.63	14021279(90.39%)

### Reliability analysis of digital gene expression sequencing data

**Fig 4:**
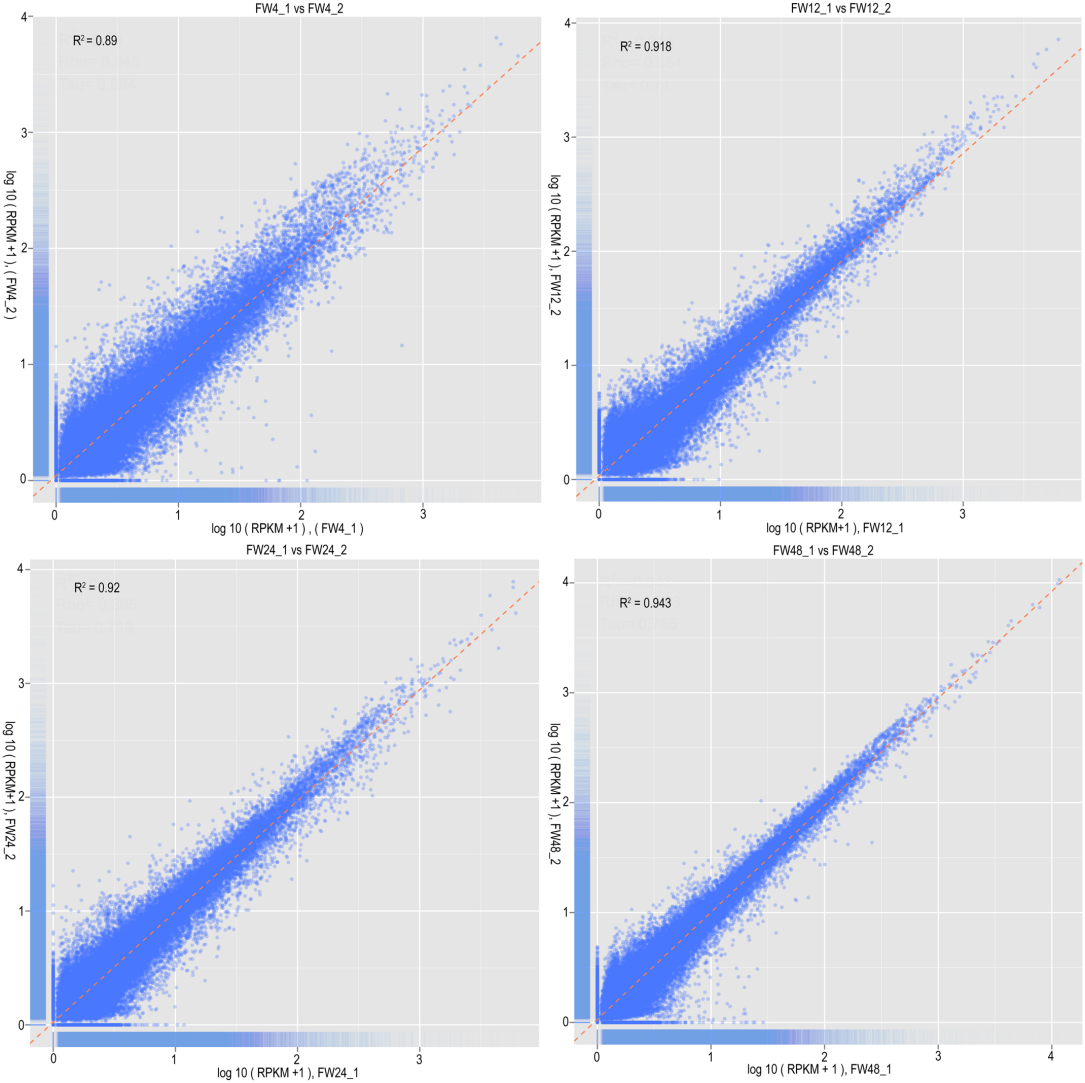
Correlation scatter diagram of gene expression among four treatment groups.

Correlation analysis of gene expression levels between replicate samples is an important technique for verifying experimental reliability and sampling accuracy. We determined the correlation coefficient between two replicate samples based on the RPKM values of genes, which were calculated using the number of read counts and the mapped gene length [25]. The R^2^ values of the four replicate groups were 0.89, 0.918, 0.92 and 0.943 (Fig 4), respectively, exhibiting remarkable consistency. These results suggest that the replicate samples had high reliability and repeatability, which ensures that digital gene expression analyses reflected actual differences in gene expression between infected and mock-infected materials.

### Analyses of DEGs

**Fig 5:**
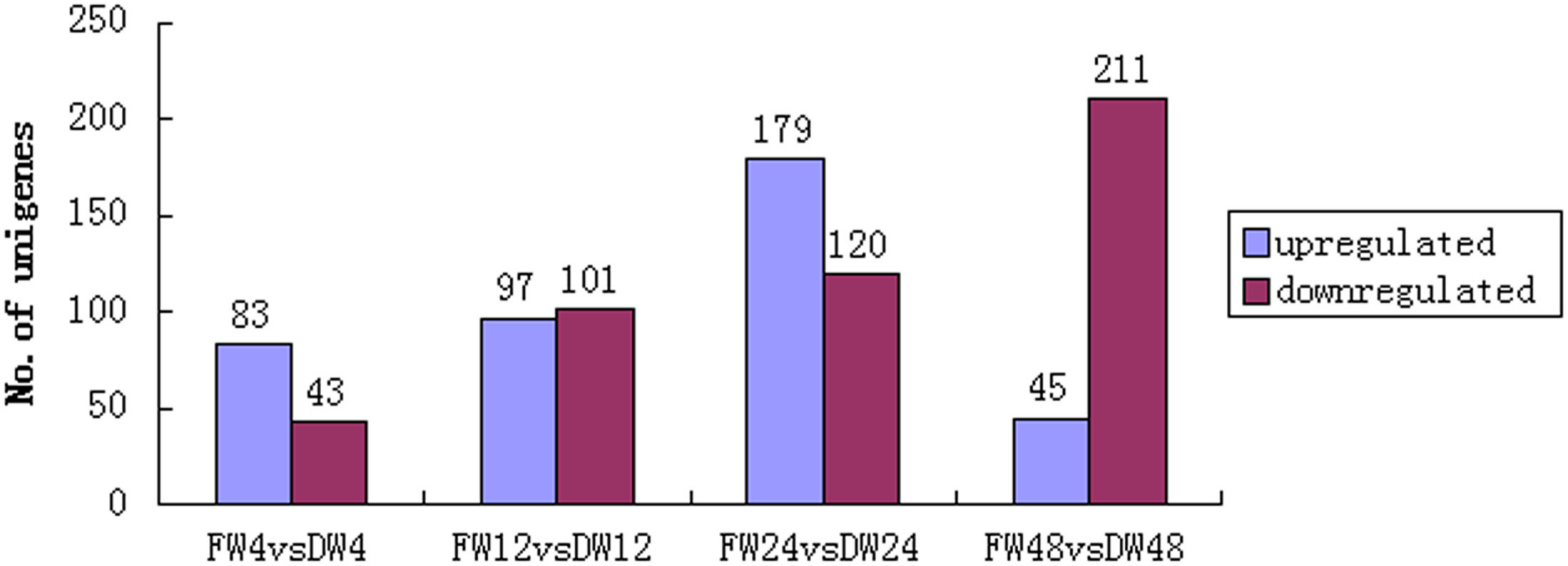
Number of differentially expressed genes at different time points after inoculation.

A total of 885 DEGs were identified between infected and mock-infected samples at each inoculation time, with a cut-off value of adjusted p-value < 0.05 and |log2 (fold change) |>1. The numbers of up- or down-regulated genes at different time points after pathogen inoculation are shown in Fig 5. As the time after inoculation increased, the number of down-regulated genes also increased, while there was a slight difference in the number of DEGs and up-regulated genes; the largest numbers of genes in these categories were detected at 24 hai.

**Fig 6:**
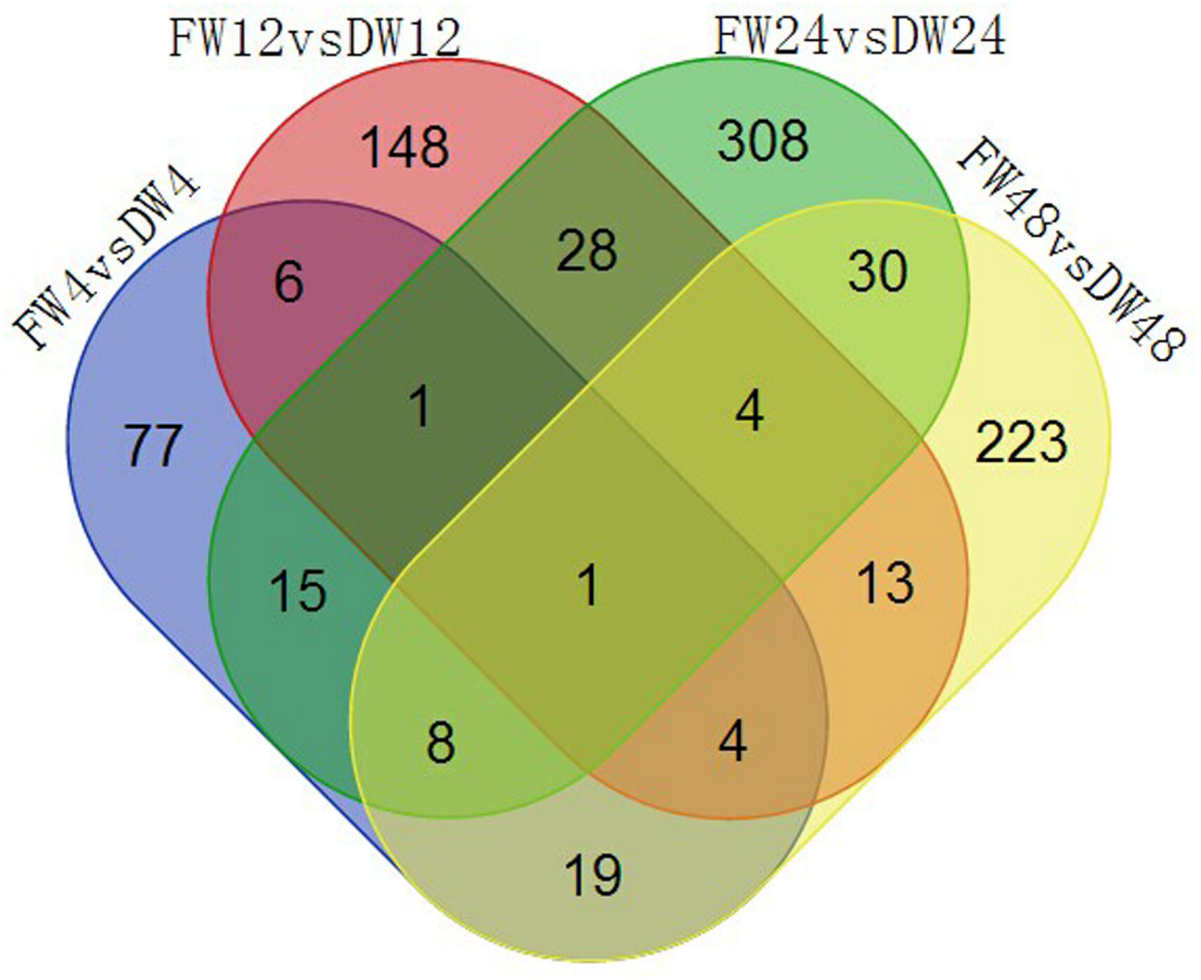
Venn diagram of differentially expressed genes.

The gene comp37314_c0 was commonly detected in the four groups (Fig 6). This gene encodes a chitinase A, indicating that chitinase plays an important role in the FOC defense system. The number of unique genes in the FW24 *vs* DW24 group was the highest as that of DEGs and up-regulated genes, suggesting that 24 hai may be a critical time in the disease resistance response in cabbage.

### Validation of RNA-seq-based DEGs by qRT-PCR

**Fig 7:**
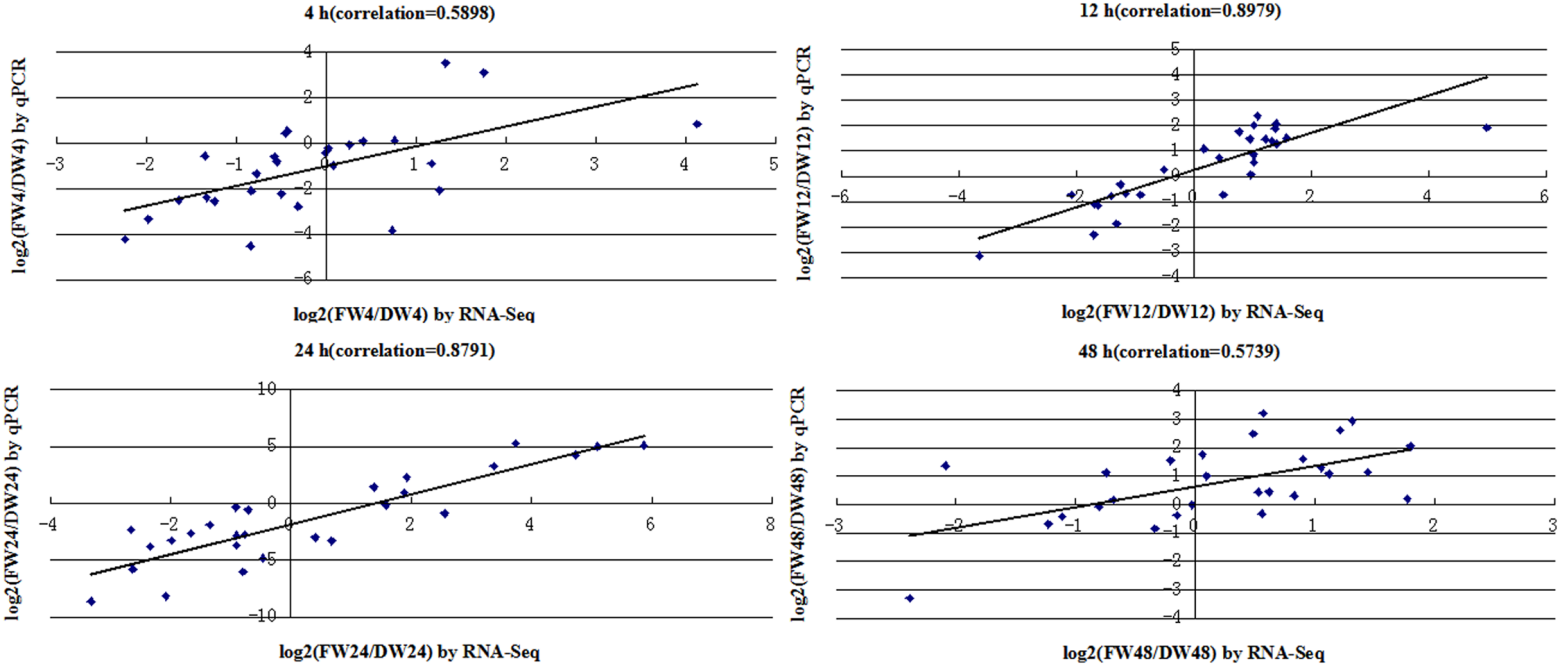
Correlation between RNA-seq and qRT-PCR data at different time points.

We selected 27 defense-related genes for validation using qPCR. The input data were relative fold changes of log2 (FW_RPKM/DW_RPKM) based on RNA-seq and log2 (2^-ΔΔCt^) based on qPCR for each DEG between infected and mock-infected roots at four stages of treatment (S1 Table), which were compared using a method described for cotton [30]. Correlation coefficients were calculated to assess the correlation between the two platforms, yielding values of 0.5898, 0.8979, 0.8791 and 0.5739 at 4 h, 12 h, 24 h and 48 h, respectively (Fig 7, correlation is significant at the 0.01 level). The tested genes displayed the same expression of up-regulation or down-regulation by the RNA-seq and qPCR, however, the fold changes varies because of the two different methods for gene quantification. So the lower correlations at 4 h and 48 h may be allowed. In summary, these results indicated that the relative expression of fold changes between the two methods showed a moderate correlation.

### Global gene regulation in response to FOC

#### MAPK signaling pathway

The MAPK signal transduction pathway is an early defense response observed upon pathogen attack. We detected seven DEGs that participate in the MAPK signaling pathway according to KEGG enrichment analysis (S2 Table), including four genes encoding heat shock 70 kDa protein 5 (HSC70b), which were significantly induced at 4 hai, and slightly regulated at 12, 24 hai and repressed at 48 hai. MAPKKK13 gene (comp57145_c0) and PDR-type ABC transporter gene (comp20414_c0) were up-regulated after pathogen infection and were induced approximately 58-fold and 5.9-fold at 24 hai, respectively. Gene (comp25940_c0) encoding lipid transfer protein (LTP) was down-regulated after pathogen infection at the four time points. GO analysis showed that comp25940_c0 participated in negative regulation of defense response and MAPK cascade. The expression patterns of most of these genes confirm the early role played by the MAPK signaling pathway in response to FOC in cabbage.

#### Calcium signaling

Calcium signaling plays important roles in triggering the biosynthesis of SA, JA and ET [31]. The PAMP (pathogen-associated molecular patterns) signaling system generates specific Ca^2+^ signals in the cytosol, and the calmodulin-binding protein CBP60g participates in activating SA biosynthesis. NO mediated by Ca^2+^ influx plays an important role in JA biosynthesis. Ca^2+^ signaling activates ACC synthase, which involved in the biosynthesis of ET. Calcium sensors and Ca^2+^-dependent protein kinases are involved in ET signaling. We detected 12 DEGs associated with calcium signaling (S2 Table). Ca^2+^ ATPase is a transport protein in the plasma membrane that regulates the amount of Ca^2+^ within a cell to transport the surrounding signal [32] Ca^2+^ ATPase gene (comp29882_c0) identified here was up-regulated at 4, 12 and 24 hai and down-regulated at 48 hai. Two calmodulin protein genes and three calmodulin-binding protein (CBP) genes were highly induced at 4 hai. Gene aquaporin TIP (tonoplast intrinsic protein, comp39047_c0) was highly induced in FW12. *MYB2* gene (comp35012_c0) was highly induced in FW4 and FW24. In addition, CBP CML37 (comp37685_c0) participates in the ET biosynthetic process, which helps confirm that Ca^2+^ signaling plays a role in ET biosynthesis. These results indicated that Ca^2+^ (as a secondary messenger) and subsequent Ca^2+^ signaling is activated during the early infection stage

#### JA, ET and SA signaling pathways

Three signaling molecules regulate signaling pathways associated with plant defense responses including JA, ET and SA. Eight genes associated with the JA pathway were identified (S2 Table), including three genes responsible for alpha-linolenic acid metabolism (a necessary pathway in JA biosynthesis) and five genes involved in JA mediated signaling pathways, including a lipid transfer protein (LTP) gene (comp25940_c0), an PDR-type ABC transporter G family gene (comp20414_c0), a CYP94A1 gene (comp44973_c0) and a myrosinase-binding protein-like (MBP) gene (comp41986_c0) and myrosinase gene (comp35843_c0). One LOX (lipoxygenase) gene (comp37329_c0), encoding a critical enzyme in the JA biosynthesis pathway, was highly induced in FW12 and FW48, whereas LTP gene (comp25940_c0) which is necessary in transduction of lipid molecules such as JA was repressed after infection, particularly in FW12 and FW24. Myrosinase gene (comp35843_c0) and JA-induced MBP gene (omp41986_c0) and were significantly down-regulated in FW12 and FW24.

Seven genes involved in SA-dependent SAR were differentially expressed (S2 Table), most of which were highly induced in FW4 and repressed in FW48 when compared to the mock-treated roots. These genes include three transcription factor genes (comp40515_c0, comp41745_c0 and comp28250_c1), two genes encoding unknown proteins (comp51677_c0 and comp33538_c0) and two genes (comp20414_c0 and comp25940_c0) participated in both SA biosynthesis and SA-dependent SAR. In addition, two UDPG genes (comp34450_c0 and comp45408_c0) that participate in the SA-mediated HR were induced at the infection roots and were highly induced in FW24 with fold change of 4.9 and 34.2, respectively (S2 Table).

Six DEGs that participate in ET-mediated signaling and four genes that function in ET biosynthesis were detected, including two genes (comp37878_c0, comp38040_c0) encoding E3 ubiquitin-protein ligase that function in the both pathways (S2 Table). All these genes were highly induced in FW4, slightly induced in FW24 and repressed in FW12 and FW48, except for UDPG (comp35166_c0), which was down-regulated in FW4 and up-regulated in the other infected roots especially in FW12 and FW24. Moreover, twenty ET-responsive transcription factor (ERF) genes were differentially expressed, most of which were highly induced at 4 hai (S3 Table). Ubiquitin-ligase genes respond to chitin, a plant-defense elicitor, play a role in the plant defense response [33]. These results suggest that the ET-mediated signaling pathway is activated upon pathogen infection, which is consistent with the early role of the ET signaling pathway in response to *F*. *oxysporum* infection in *Arabidopsis* [14].

#### Lignin biosynthesis

In many cases, during plant-pathogen incompatible interactions, transcriptional profiling studies have revealed genes involved in the biosynthesis and modification of cell wall components [30]. Lignin deposited during plant-pathogen interactions forms a physical barrier against infection. In this study, we identified 11 DEGs that participate in phenylpropanoid-lignin pathway (S2 Table), including key enzymes in lignin biosynthesis: peroxidase (POD, comp34233_c0, comp37399_c0, comp28296_c0, comp16630_c0), ferulic acid 5-hydroxylase (F5H, comp35066_c0), caffeic acid 3-O-methyltransferase (COMT, comp30914_c0) and pyridoxal-phosphate dependent enzyme (comp35737_c0), glycosyl hydrolase (comp35141_c0, comp30472_c0,comp35843_c0), as well as a gene that participates in xylem and phloem pattern formation (comp44045_c0). Three POD genes and genes encoding glycosyl hydrolase and pyridoxal-phosphate-dependent enzyme involved in the biosynthesis of L-phenylalanine (intermediate of lignin biosynthesis) were significantly repressed in FW4, FW12 and FW24 but induced in FW48. Another POD gene (comp28296_c0) was highly induced in FW12 and FW48 but repressed in FW4 and FW24. F5H gene (comp35066_c0) was induced in FW12, FW24 and FW48. The COMT gene (comp30914_c0) was significantly repressed after pathogen inoculation. Overall, these regulated genes indicate that the lignin biosynthesis pathway is activated during the defense response to FOC. In addition, myrosinase gene (comp35843_c0) identified here participates in both the phenylpropanoid pathway and the JA-mediated pathway, suggesting that the two pathways interact in the disease resistance.

#### Transcription factors (TFs)

Defense-related genes are normally regulated by TFs, which play direct or indirect roles in different signaling pathways [14]. Among the 885 DEGs, a total of 47 differentially regulated TF genes were identified (S3 Table), including genes encoding WRKY (1), MYC (1), CxHy zinc finger (4), bHLH (4), NAC (4), MYB (5), HSF (8) and ERF (20). Most TF genes were highly up-regulated at 4 hai but down-regulated at 12, 24 and 48 hai.

#### Protein kinases

Protein phosphorylation and dephosphorylation are important steps in many signal transduction pathways. Protein kinases involved these process help complete the signal transmission. We identified eight differentially expressed protein kinase genes associated with the defense system (S4 Table), including genes encoding MEKK (1), serine/threonine protein kinase (3), leucine-rich repeat (LRR) transmembrane protein kinase (2) and lectin protein kinase (2). The MEKK gene (comp57145_c0) was induced in all infected roots and up-regulated about 58.2 fold at 24 hai. One LRR-type kinase gene (comp19030_c0) was up-regulated at 12, 24 and 48 hai and significantly up-regulated at 24 hai.

#### Detoxifying-related proteins

UDPG [34] and the PDR-type ABC transporters [35] detoxify deoxynivalenol (DON), a virulence factor, during infection by Fusarium species. In this study, we detected DEGs encoding ten UDPG and three PDR-type ABC transporters (S5 Table). All the genes were highly induced in FW24 except for two UDPG genes (comp29479_c1, comp5003_c0).

#### Defense genes respond to FOC infection

PR genes are important in disease resistance. The antifungal thaumatin-like protein (PR5) cause osmotic breakage of transmembrane pores on the fungal plasma membrane; the Bet v I family protein (PR10) are involved in the synthesis of compounds such as antibiotics [36]. The chitinase family PR3 hydrolyze the β-1,4 glycosidic linked to the N-acetylglucosamine residues of chitin to participate in the disease resistance [37]. Six PRs were differentially expressed (S6 Table), including one *PR5* gene (comp35815_c0), four *PR10* genes (comp17904_c0, comp17904_c1, comp20529_c0, comp20089_c0) and one *PR3* gene (chitinase, comp21138_c0). *PR5* gene was highly induced in FW12 and FW48 and repressed in FW4 and FW24. *PR10* gene (comp20089_c0) was induced in FW4 and FW12 and repressed in FW24 and FW48. The other genes were all down-regulated after inoculation.

Chitinases, which function as hydrolytic enzymes in plants, break down chitin (the major component of the cell walls of fungi) into mono- and oligomers to destroy the fungi and prevent fungal infection. Studies supported that chitinases were antifungal to inhibit growth of fungal hyphae [38, 39]. Four genes encoding chitinase were identified by DGE analysis (S6 Table). Among them, comp37238_c0 and comp37314_c0 were highly induced in all inoculated samples, Comp32071_c0 and comp37179_c0 were only induced in FW12.

Cytochrome P450 genes were improved to have a role in response to *F*. *oxysporum* infection in *Arabidopsis* [14]. Five cytochrome P450 genes involved in JA, lignin and indole glucosinolate pathways, which are required for the innate immune response, were differentially expressed (S6 Table). comp43877_c0 (CYP82F1), which is involved in the indole glucosinolate metabolic process, was repressed in infected roots except for FW48. comp120471_c0 (CYP71A12), which participates in induced systemic resistance, was highly induced in FW24 and highly repressed in FW48. For genes involved in JA-metabolic process, comp31152_c0 (CYP94B1) was induced in all inoculated samples except for FW24 and comp44973_c0 (CYP94C1) was down-regulated in all inoculated samples. comp35066_c0 (F5H, CYP84A1) involved in lignin biosynthesis was discussed above.

Glucosinolate metabolism is a required component of the plant defense response against microbial pathogens [40]. The hydrolysis of glucosinolates is catalyzed by myrosinases, forming nitrile, isothiocyanate, amine, epithionitrile, thiocyanate, oxazolidine-2-thione and so on [41]. In Brassicales plants, the glucosinolate-myrosinase defense system produces toxic volatile compounds during pathogen attack [42], and several products have demonstrated in vitro toxicity to mycelial growth in cereal root pathogens [43]. Certain types of MyAPs (myrosinase-associated proteins), the ESPs (epithiospecifiers), function as myrosinase cofactors, which are required to modulate the specificity of myrosinases towards the production of particular enzyme products [44]. MBPs (myrosinase-binding proteins) may bind to carbohydrates present in fungal pathogens through their lectin domain to enhance their defense reactions [45]. One myrosinase gene (comp35843_c0), two MyAP genes (comp38098_c0, comp19088_c0) and three MBP genes (comp32249_c0, comp32249_c1, comp41986_c0) were down-regulated in all the infected roots (S6 Table). Results implied a role of these genes in response to FOC infection.

### Expression profiles of DEGs selected in R4P1 and R2P2 by qRT-PCR

**Fig 8:**
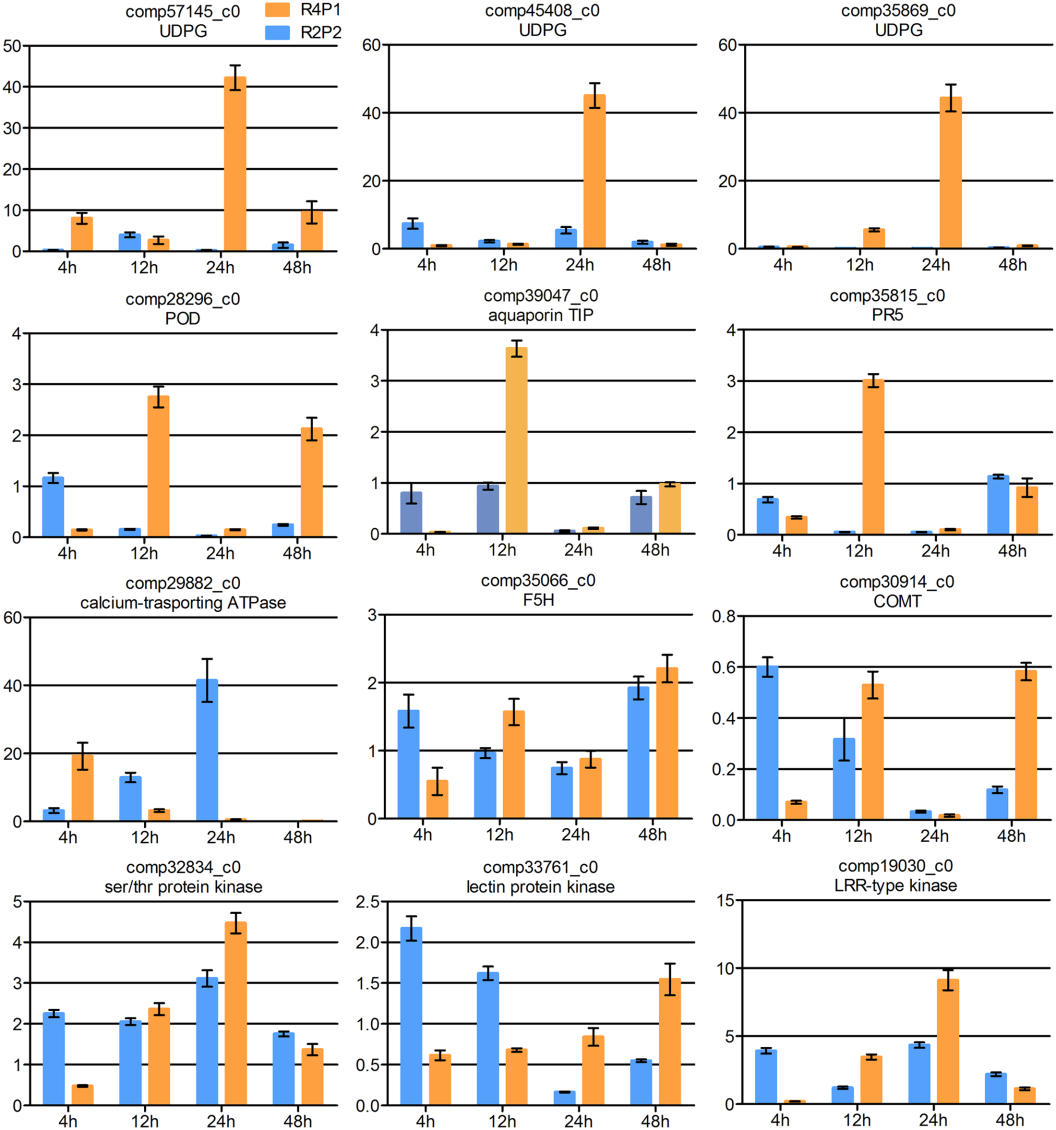
Expression profiles of DEGs in R4P1 and R2P2 by qRT-PCR. The expression levels on the y-axis were relative to non-inoculated sample from each genotype after normalization with *GAPDH* gene. Error bars represent the SD for two independent experiment, and three technical replicates.

Some of the DEGs were selected to characterize the gene expression profiles between R4P1 (resistant) and R2P2 (susceptible) after FOC inoculation by qRT-PCR (Fig 8). Data were shown in S8 Table. MAPKKK13 (comp57145_c0) and UDPG (comp45408_c0, comp35869_c0) were highly induced more than 40-fold change in R4P1 at 24 hai, while showed low expression level at other times in R4P1 and in R2P2. POD (comp28296_c0), aquaporin TIP (comp39047_c0) and PR5 (comp35815_c0) displayed high expression level at 12 hai or 48 hai but were down-regulated at other times in R4P1 and in R2P2. Calcium-binding ATPase (como29882_c0) showed high expression level at 4 hai in R4P1 compared to R2P2. The expression patterns of aquaporin TIP and Calcium-binding ATPase validated the early role of calcium signaling played in resistance to FOC in cabbage. The expression of the other genes showed an increase trend in R4P1 and a decrease trend in R2P2, and most genes showed a higher expression level in R4P1 except for the 4 hai. These results indicated that these DEGs play roles in disease resistance to FOC in cabbage.

## Discussion

### Examining the transcriptomes of R4P1 roots during FOC infection provides comprehensive knowledge for FOC resistance-related gene discovery

In this study, using Illumina sequencing, we identified 62,506 unigenes from R4P1 roots, 79.92% of which were identified in at least one database. The presence of unigenes that could not be annotated might be due to the relatively short lengths of their assembled sequences. It is particularly notable that the number of assembled unigenes (62,506) does not match the 45,758 genes in the recently released cabbage genome sequence. Reasons are the following: (1) most of unigenes obtained were parts of corresponding genes; (2) some were different regions from the same gene; (3) some were discarded from further analysis because of their short size or unsatisfactory alignment [16]. Nonetheless, the transcriptome data offered an overview of the gene expression profiles of FOC-inoculated roots of R4P1 and a valuable set of genes with which to investigate FOC resistance-related genes.

### MAPK signaling pathway functions in the response to FOC

Recognition of bacterial elicitor flg22 by receptor kinase FLS2 in *Arabidopsis* activates the cascade reaction MEKK1-MKK4/MKK5-MPK3/MPK6-WRKY22/WRKY29 [14, 46]. The MKK4/MKK5-MPK3/MPK6 cascade in *Botrytis cinerea*-infected Arabidopsis plants regulates the biosynthesis of camalexin, which is the major phytoalexin in *Arabidopsis* [47]. In the current study, however, no such MAPK cascade associated with the defense response to FOC was detected, which is consistent with the results of a study in *Arabidopsis*, cotton and tomato [48]. However, we identified seven genes that participate in MAPK signaling pathway in this study. Among them, four Hsp70b genes were annotated in BP description as viral process or response to virus to trigger the MAPK signaling pathway, which indicated that HSC70b proteins were upstream in MAPK signaling pathway. Indeed, the expression of two *HSC70* isoforms were upregulated by pathogen infection, while loss-of-function mutants of individual cytosolic *HSC70* genes do not display defense phenotypes [49] LTP genes (comp25940_c0) and PDR-type ABC transporter gene (comp20414_c0) participating in MAPK cascade to negatively regulate the defense response function downstream of this pathway. Although only one MAKKK13 (MEKK13) gene was identified, the analysis above implied that there may be another MAPK cascade in FOC resistance in cabbage.

### SA-dependent pathways function in FOC resistance in *Brassica oleracea*

During the disease resistance response, SA triggers the expression of a number of genes, which can be divided into two groups: group 1, genes that function immediately/early in the HR that were independent of *NPR1* (*nonexpresser PR genes 1*), such as those encoding glycosyltransferases and glutathione S-transferases; group 2, genes that function late in the SAR, such as PRs, which require *NPR1* induction [50]. The HR occurs at the site of attack, where *NPR1* is degraded to remove its inhibitory effect on effector-triggered cell death and defense, but it accumulates in neighboring cells to promote cell survival and SA-mediated resistance [51], indicating that *NPR1* is not involved in the HR.

We identified two differentially expressed SA-induced UDPG genes (comp45408_c0 and comp34450_c0), whose induction by SA is independent of *NPR1*. In addition, comp34450_c0 is homologous with pathogen-inducible *AtSGT1*, whose expression is an early disease response in *Arabidopsis* that may be involved in the accumulation of glucosyl SA during the disease process [52]. SGT1 is an early, essential component of *R* gene-triggered disease resistance and can interact with the LRR domains of certain NBS-LRR proteins [53–55]. Furthermore, the interaction between SGT1 and HSC70 regulates the immune responses in *Arabidopsis* [49], and in this study, we identified four *HSC70* genes that function in the MAPK signaling pathway, which suggests that crosstalk might occur between the MAPK signaling pathway and the SA-UDPG-dependent HR pathway.

In this study, our results indicate that SA-dependent SAR is involved in cabbage defense response against FOC. However, there is no evidence that these genes are involved in the EDS/PAD4-SA-NPR1-PR signaling pathway. In addition, *F*. *oxysporum* is considered to be a hemibiotrophic pathogen, as it begins its infection cycle as a biotroph and subsequently becomes a necrotroph [15]. The SA-dependent defense pathway is activated in the defense response against biotrophic pathogens or in the biotrophic phase [17]. Most genes that participate in SA-dependent SAR were highly induced in FW4, FW12 and FW24 and repressed in FW48, indicating that 48 hai is likely to be the turning point between the biotrophic phase and the necrotrophic phase of the FOC in R4P1 in this study.

Notably, the candidate FOC resistance gene (comp30068_c1) identified in this study (S6 Table), encoding a TIR-NB-LRR-type resistance gene, annotated as *SNC1* (*Suppressor of NPR1-1*, *Constitutive1*), is involved in SA-dependent SAR. Mutant plants constitutively express PR genes, and PRs in the SA-mediated SAR require *NPR1*, which function downstream of SA[51]. *EDS1* (*enhanced disease susceptibility 1*), *PAD4* (*phytoalexin deficient 4*) and *MOS3* (*modifier of SNC1*,*3*) are required in *SNC1* resistance signaling. *EDS1*, an essential component of resistance specified by TIR-NB-LRR, functions upstream of SA-dependent defense responses and acts as a pathogen effector target in *Arabidopsis* [56]. EDS1 interacts with its positive co-regulator PAD4, resulting in mobilization of the SA defense pathway in rice and *Arabidopsis* [57]. Studies in rice and Arabidopsis suggest that EDS1 and PAD4 form a dimeric protein complex, which might be important for triggering the SA signaling pathway in plants [58, 59]. The analysis also suggested that the TIR-NB-LRR-EDS/PAD4-SA-NPR1-PR pathway may function in the defense response of cabbage to FOC. Moreover, study verified that *R* gene-mediated resistance associated with activation of an SA-dependent signaling pathway induces the expression of certain PR proteins, which contribute to resistance in *Arabidopsis* [17]. However, the classic marker genes of this pathway, including the candidate resistance *R* gene, *EDS1*, *PAD4* and *NPR1*, were not differentially expressed after inoculation, perhaps because these genes were constitutively expressed in cabbage roots or because, this pathway may specifically function in foliar tissues, as observed in *Arabidopsis* [60]. Therefore, further research is required to determine the exact mode of action of SA-mediated fusarium wilt resistance in cabbage. Nonetheless, the results indicate that two SA-mediated signaling pathways are involved in the disease resistance system, including SA-mediated HR and SA-mediated SAR.

### The JA-mediated signaling pathway plays an important role in FOC resistance in cabbage

JA signaling pathway is important for disease resistance, which is distinct from the classic SA-dependent SAR [61]. JA biosynthesis and JA-mediated signaling pathways are important components of the fungal resistance system in plants [12–14, 30]. Our data demonstrate that the JA-mediated signaling pathway also contributes to FOC resistance in cabbage. Myrosinase gene (comp35843_c0) is homologous to *Arabidopsis* gene *TGG1 (Thioglucoside glucohydrolase1)*, whose expression and activity are controlled by COI1 [62]. JA-induced MBP (comp41986_c0) gene depends on *COI1* for expression [63]. COI1 is a key player in JA perception and JA-mediated transduction pathway, although *COI1* was not differentially expressed in this study. It implied that Myrosinase gene (comp35843_c0) and JA-induced MBP (comp41986_c0) gene identified here were downstream of the JA-mediated signaling. Myrosinases, Brassicaceae-specific β-glucosidases that interact with MBPs, are responsible for the hydrolysis of glucosinolate defense compounds in Brassica crops [46, 64]. When plants are infected with fungi, the non-toxic glucosinolates are hydrolyzed by myrosinases into toxic compounds for disease resistance, including isothiocyanates, thiocyanates, nitriles and epithionitriles [65]. Due to the important role played by the Brassicaceae-specific glucosinolates-myrosinase system in fungal defense, the identification of myrosinase and *MBP* genes helps confirm that the JA signaling pathway plays significant role in FOC resistance in cabbage. Moreover, MBP (comp41986_c0) is associated with *Arabidopsis RIN4* (*RPM1-interacting protein 4*), which is a negative regulator of plant immunity, and proteolytic cleavage and phosphorylation of which by bacterial effectors activates two RIN4-associated R proteins (RPS2 and RPM1) resulting in the ETI (effector-triggered immunity) defensive reaction and further restriction of colonization by pathogens [14, 66]. This result highlights the function of MBP (comp41986_c0) in disease resistance in cabbage.

In addition, the PDR-ABC transporter gene comp20414_c0 and the lipid-transfer protein gene comp25940_c0 are involved in the MAPK cascade, as well as JA- and SA-mediated signaling pathways, indicating that these genes participate in disease resistance to FOC in cabbage by sharing different signaling pathways. Indeed, in cucumber, the expression of *CsPDR12* significantly increases upon the addition of JA, SA or ABA, suggesting that CsPDR12 is involved in a phytohormone-mediated response of plants to different stimuli by sharing different signaling pathways [67].

**Fig 9:**
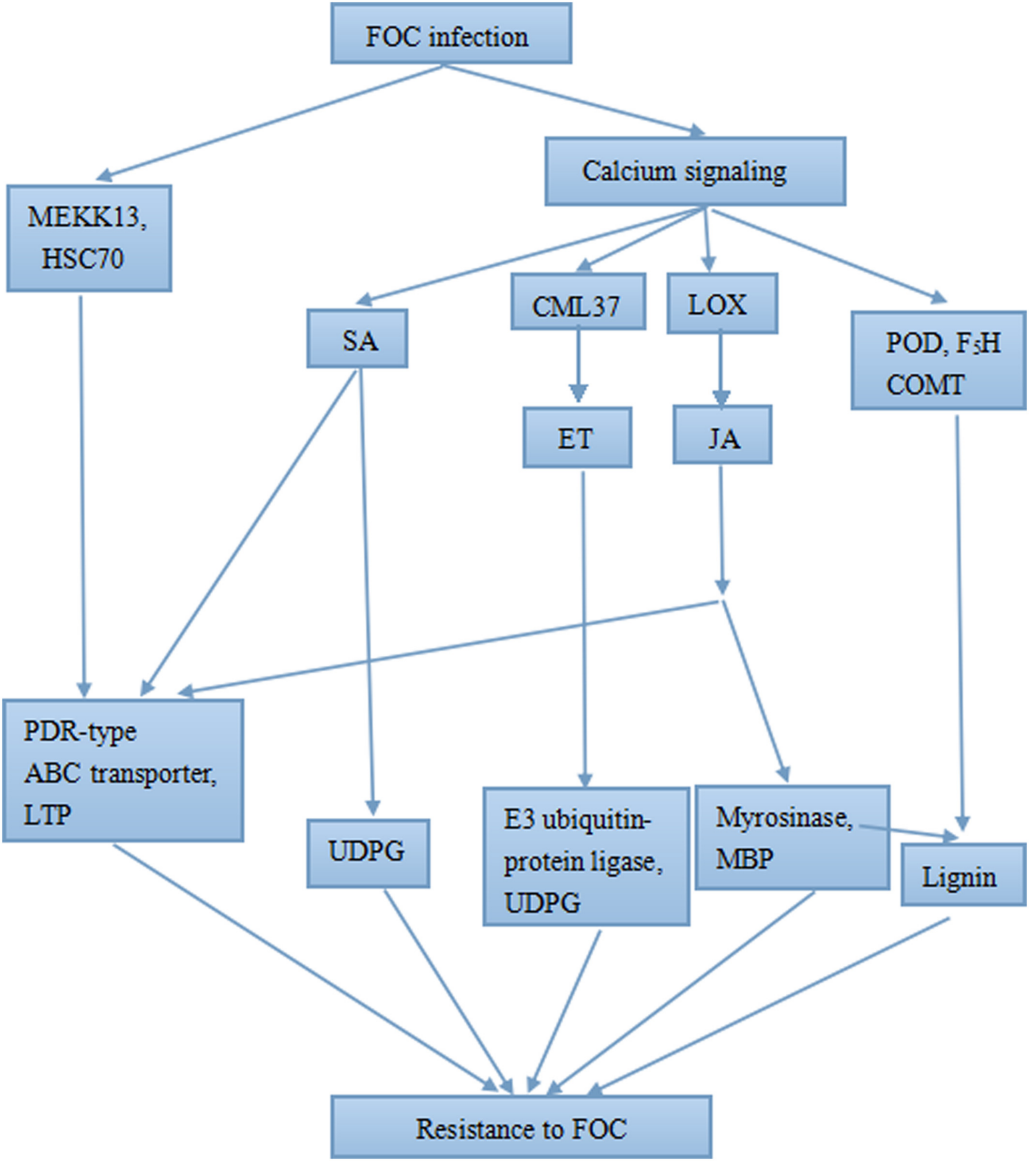
Schematic representation of the response of R4P1 to FOC infection. MEKK13: MAPKKK13; HSC70: heat shock 70 kDa protein; LTP: lipid-transfer protein; MBP: myrosinase-binding protein.

Much is known about the ET-, SA- and JA-mediated signaling systems [68], which allowed us to construct a schematic diagram describing the FOC resistance response in R4P1(Fig 9).

### Resistance genes in response to FOC in cabbage

In this study, many NBS-LRR genes were identified, including 213 TIR-NBS-LRR genes and 65 CC-NBS-LRR genes (S7 Table). These genes were not differentially expressed in inoculated roots compared to mock-inoculated roots under the filter conditions, and most of them were suppressed after infection, which is consistent with the results of a study in bananas, which showed that the expression of most NBS-LRR resistance proteins were quite low in resistant plants [13]. The slight downregulation of the candidate resistance gene comp30068_c1 in all the infected roots may be the result of the interaction of *R* gene-pathogen, which activates defense responses. Studies showed that there were insertion/deletion variations of the FOC-resistance gene between resistant cabbage material and susceptible materials [9, 11]. The results indicated that the *R* genes displayed constitutive expression in resistant material.

In conclusion, our transcriptome data provide a comprehensive overview of the gene expression profiles at the four stages of FOC infection, which will facilitate further analysis of the molecular mechanism underlying FOC resistance in cabbage. We proposed a putative network underlying this response in R4P1 (Fig 9). Early defense systems, including calcium signaling, MAPK signaling and SA-UDPG HR, were activated, and SA-dependent SAR, JA- and ET-mediated pathways and the lignin biosynthesis pathway were activated as well, suggesting that they play significant roles in FOC resistance in cabbage. Finally, our results suggest that these signaling pathway are not independent, instead interacting, which demonstrates that R4P1 utilizes different, effective defense pathways comprising a complex resistance network in response to FOC infection.

## Supporting Information

S1 Table Primers used in qPCR of selected genes and relative fold changes based on RNA-seq and qPCR. (XLS)

S2 Table Pathways involved in the defense system and detailed expression patterns and annotations of related genes. (XLS)

S3 Table Detailed expression patterns and annotations of transcription factors related to the defense system. (XLS)

S4 Table Detailed expression patterns and annotations of protein kinases related to the defense system. (XLS)

S5 Table Detailed expression patterns and annotations of detoxifying-related proteins participating in the defense system. (XLS)

S6 Table Detailed expression patterns and annotations of defense-related genes. (XLS)

S7 Table Detailed expression patterns and annotations of NBS-LRR genes. (XLS)

S8 Table Results of genes selected in R4P1 and R2P2 by qRT-PCR. (XLS)
